# Magnetic susceptibility anisotropy in normal appearing white matter in multiple sclerosis from single-orientation acquisition

**DOI:** 10.1016/j.nicl.2022.103059

**Published:** 2022-05-28

**Authors:** Renat Sibgatulin, Daniel Güllmar, Andreas Deistung, Christian Enzinger, Stefan Ropele, Jürgen R. Reichenbach

**Affiliations:** a Medical Physics Group, Institute of Diagnostic and Interventional Radiology, Jena University Hospital — Friedrich Schiller University Jena, Philosophenweg 3, 07743 Jena, Germany; b University Clinic and Outpatient Clinic for Radiology, Department for Radiation Medicine, University Hospital Halle (Saale), Ernst-Grube-Str. 40, 06120 Halle (Saale), Germany; c Department of Neurology, Medical University of Graz, Auenbruggerplatz 22, 8036 Graz, Austria; d Michael Stifel Center Jena for Data-Driven and Simulation Science, Friedrich-Schiller-University Jena, Jena, Germany

**Keywords:** AMS, apparent magnetic susceptibility, AO, age at onset, DD, disease duration, DWI, diffusion-weighted imaging, EDSS, expanded disability status scale, EPI, echo-planar imaging, FA, fractional anisotropy, GCC, genu of the corpus callosum, GRE, gradient recalled echo, HC, healthy control, HDI, highest density interval, LL, lesion load, MAP, maximum a posteriori (estimate), MCMC, Markov chain Monte Carlo, MD, mean diffusivity, MS, multiple sclerosis, MSA, magnetic susceptibility anisotropy, NAWM, normal appearing white matter, ODF, orientation distribution function, OR, optic radiation, PQ, peak quotient, QSM, quantitative susceptibility mapping, RD, radial diffusivity, ROI, region of interest, RRMS, relapsing-remitting multiple sclerosis, SCC, splenium of the corpus callosum, SLF, superior longitudinal fascicle, ST, susceptibility tensor, TA, acquisition time, TE, echo time, TR, repetition time, WM, white matter, Multiple sclerosis, Quantitative susceptibility mapping, Orientation dependence, White matter, Magnetic susceptibility anisotropy, Tissue microstructure

## Abstract

Quantitative susceptibility mapping (QSM) has been successfully applied to study changes in deep grey matter nuclei as well as in lesional tissue, but its application to white matter has been complicated by the observed orientation dependence of gradient echo signal. The anisotropic susceptibility tensor is thought to be at the origin of this orientation dependence, and magnetic susceptibility anisotropy (MSA) derived from this tensor has been proposed as a marker of the state and integrity of the myelin sheath and may therefore be of particular interest for the study of demyelinating pathologies such as multiple sclerosis (MS). Reconstruction of the susceptibility tensor, however, requires repeated measurements with multiple head orientations, rendering the approach impractical for clinical applications.

In this study, we combined single-orientation QSM with fibre orientation information to assess apparent MSA in three white matter tracts, i.e., optic radiation (OR), splenium of the corpus callosum (SCC), and superior longitudinal fascicle (SLF), in two cohorts of 64 healthy controls and 89 MS patients. The apparent MSA showed a significant decrease in optic radiation in the MS cohort compared with healthy controls. It decreased in the MS cohort with increasing lesion load in OR and with disease duration in the splenium. All of this suggests demyelination in normal appearing white matter. However, the apparent MSA observed in the SLF pointed to potential systematic issues that require further exploration to realize the full potential of the presented approach. Despite the limitations of such single-orientation ROI-specific estimation, we believe that our clinically feasible approach to study degenerative changes in WM is worthy of further investigation.

## Highlights

• Orientation dependence of QSM is studied in a large cohort of MS patients.
• Apparent magnetic susceptibility anisotropy (MSA) obtained from single-orientation QSM.
• Apparent MSA found decreased in optic radiation (OR) of MS patients.
• Apparent MSA decreases with lesion load in OR and with disease duration in splenium.
• Negative apparent MSA observed in SLF indicates limitations of the proposed method.

## 1 Introduction

Multiple sclerosis (MS) is a chronic inflammatory demyelinating disease of the central nervous system (Frischer et al., 2009). Its pathological hallmark is the presence of focal lesions in the white matter (WM) of the brain and spinal cord associated with myelin loss, axonal injury, and inflammation (Lassmann, 2008). Such WM lesions are routinely assessed by magnetic resonance imaging (MRI) for both diagnosis and disease monitoring. There is increasing evidence that diffuse inflammatory processes also occur in the radiologically normal-appearing white matter (NAWM) (Comi et al., 2004, Granberg et al., 2017), associated with impaired iron metabolism (Hametner et al., 2013) and demyelination (Liu et al., 2015, Rahmanzadeh et al., 2021). Specific contrast techniques in MRI, such as phase gradient echo (GRE) imaging and quantitative susceptibility mapping (QSM), have shown delicate sensitivity to both iron accumulation (Schweser et al., 2010, Langkammer et al., 2012) and myelin sheath status (Liu et al., 2011, Schweser et al., 2011, Schweser et al., 2012, Lee et al., 2012, Li et al., 2012a). In particular, QSM has been used extensively to assess magnetic susceptibility of MS lesions and perilesional tissue (Chen et al., 2014) as well as iron deposition in grey matter (Langkammer et al., 2013). However, applying this method to WM is challenging due to the apparent orientation dependence of the recovered susceptibility (Lee et al., 2010, Denk et al., 2011). In this context, the magnetic susceptibility tensor (ST) has been proposed as an explanation for this orientation dependence of the scalar apparent magnetic susceptibility (AMS) (Lee et al., 2010), leading to the development of a number of approaches to estimate this tensor (Liu, 2010, Li et al., 2012b, Wisnieff et al., 2013) and to the proposition that the orientation dependence of the apparent magnetic susceptibility, $\chi_{app}$⁠, varies proportionally to the sine square of the fibre-to-field angle (Li et al., 2012a).

Upon minor transformations (see appendix A of Sibgatulin et al., 2021), this angular dependence can be expressed as.

(1) $$\chi_{app}\left(\theta \right)=\left(\chi_{\|}-\chi_{⊥}\right){\text{cos}}^{2}\theta +\chi_{0},$$

where θ is the fibre-to-field angle, $\chi_{\|}$ and $\chi_{⊥}$ are components of the radially symmetric assumed fibre susceptibility tensor, and $\chi_{0}$ is a term that subsumes all orientation-independent contributions to AMS, including any orientation-independent contributions from myelin. Notably, W. Li et al. proposed that both $\chi_{\|}$ and $\chi_{⊥}$ are proportional to the volume fraction of myelin lipids and that the difference $\chi_{\|}-\chi_{⊥}$ — termed magnetic susceptibility anisotropy (MSA) — can serve as a marker for myelination (Li et al., 2012a).

Importantly, magnetic susceptibility anisotropy is a property of the susceptibility tensor at a given location (e.g., voxel) and as such is only accessible when AMS is obtained at the same position for different orientations of the fibre with respect to the main magnetic field. For applications where only a single head orientation acquisition is possible, we propose to estimate the *apparent* MSA (δχ) in an entire white matter tract by using the inherent distribution of fibre-to-field angles in that tract to formulate the estimation of δχ as a regression problem (along with the isotropic apparent magnetic susceptibility $\chi_{iso}$⁠, which is a tract-averaged counterpart of $\chi_{0}$):

(2) $$\chi_{app}\left(\theta \right)=\delta \chi \;{\text{cos}}^{2}\theta +\chi_{iso}.$$

We anticipate that in this way, at least some of the microstructural changes associated with MS that are expected to manifest biophysically in the components of the local susceptibility tensor can be captured with clinically feasible imaging while using a scanning protocol that is tolerable for patients. If such a simplified approach to a macroscopic description of what are indeed complicated processes at the molecular level is able to reliably target important microstructural changes associated with this disease, this could become a sensitive marker that proves useful in the clinical context.

To reiterate our approach, in contrast to Equation (1), the relationship between $\chi_{app}$ and δχ in Equation (2) is thus not defined on a voxel basis using the voxel-specific MSA value, but in the least squares sense, where δχ and $\chi_{iso}$ are considered as regression coefficients of the relationship in Equation (2) applied to the complete white matter tract under consideration.

In a previous work (Sibgatulin et al., 2021), the applicability of the model underlying Equation (2) was assessed in several tract-based white matter regions-of-interest (ROIs) in a cohort of healthy subjects. Although AMS showed an unexpected dependence on the fibre-to-field angle in some ROIs (likely due to the ill-conditioned dipole inversion process), the apparent magnetic susceptibility in at least two ROIs — i.e., optic radiation (OR) and the splenium of corpus callosum (SCC) — agreed well with previously reported results (Li et al., 2012b, Wisnieff et al., 2013, Zhang et al., 2021). Given that the limits of applicability even for the voxel-wise model in Equation (1) have not yet been fully clarified, this agreement is taken as an indication of the applicability of the ROI-based model to the two aforementioned ROIs. In contrast, the observation in the superior longitudinal fascicle (SLF) did not clearly match the current expectation. However, due to the robustness of the result, SLF is included in this work for completeness and as a potential counterexample.

In the present work, we aim to apply our approach (Equation (2)) in a cohort of patients with MS to assess both apparent magnetic susceptibility anisotropy (δχ) and isotropic apparent magnetic susceptibility ($\chi_{iso}$). Based on our previous analysis in healthy subjects, we focused on three anatomic regions of interest: OR, which showed robustly positive δχ in all healthy subjects studied; SLF, which demonstrated remarkably robust but unexpectedly negative δχ; and SCC, which showed positive δχ values in all but a few subjects. Both the tensor-derived MSA, $\left(\chi_{\|}-\chi_{⊥}\right)$ (Equation (1)), and the *apparent* MSA, δχ (Equation (2)), have been proposed as markers for myelination (Li et al., 2012a), with the latter successfully applied to corticospinal tracts in a small cohort of paediatric cerebral palsy patients (Zhang et al., 2021). However, to our knowledge, neither apparent MSA (δχ) nor tensor-based MSA have been used to study NAWM in a large cohort of MS patients, presumably due to the significant experimental inconvenience associated with tensor-based MSA and the challenge of estimating apparent MSA. As indicated above, we hypothesise that δχ reflects previously reported demyelination in the NAWM of MS patients (Enzinger et al., 2015, Rahmanzadeh et al., 2021), similar to what is hypothesized for tensor-derived MSA (Li et al., 2012a). The change in isotropic AMS in patients is less easy to predict, as demyelination should lead to an increase in $\chi_{iso}$ (i.e., to a less diamagnetic $\chi_{iso}$), whereas decreased iron levels (Hametner et al., 2013) may lead to a decrease in $\chi_{iso}$ (i.e., to a less paramagnetic $\chi_{iso}$) compared with the matched control cohort.

Thus, the main objective of this work is essentially to demonstrate that apparent MSA is able to capture potential microstructural changes in specific NAWM regions in patients with MS.

A note to the reader: In the following, the terms *apparent magnetic susceptibility anisotropy*, *apparent MSA*, and δχ are used interchangeably. The same applies to *apparent magnetic susceptibility*, *AMS*, and $\chi_{app}$; and finally to *isotropic apparent magnetic susceptibility*, *isotropic AMS*, and $\chi_{iso}$.

## 2 Methods

### 2.1 Cohorts

The study included a cohort of 64 volunteers without known neurological conditions (aged 24–66 years, mean age 35 years, 36 female) and a cohort of 89 patients with relapsing-remitting MS (RRMS) (aged 19–65 years, mean age 38 years, 64 female) who underwent a routine examination at the Department of Neurology, Medical University of Graz, using a predefined MRI protocol (see below). Clinical disability was assessed using the expanded disability status scale EDSS (Kurtzke 1983) and ranged from 0 to 5 with a median score of 1. Disease duration spanned 1 to 32 years with a median of 9 years (see Supplementary Figure S1). All subjects were examined once, and the images were subjected to visual quality assessment. The two cohorts were recruited and scanned at two different sites with identical MRI systems, imaging protocols, equipment and software versions. In accordance with the Declaration of Helsinki, the study was approved by the appropriate local ethics committees, and all participants provided written informed consent.

### 2.2 Data acquisition and processing

MRI data were acquired on 3 T MRI systems (Siemens Prisma) using a 20-channel head-coil. A detailed description of data acquisition and processing has been published previously (Sibgatulin et al., 2021) and is briefly summarized below.

Two 3D multi-echo gradient-echo (GRE) sequences were acquired with flip angles of 6° (PDw) and 35° (T1w), TR = 37 ms, TE_1–5_ = 8.12–29.4 ms, ΔTE = 5.32 ms, and voxel size of (1 × 1 × 1) mm^3^, TA = 8 min 26 s, FOV = LR: 168 mm, PA: 224 mm, IS: 192 mm. Diffusion properties were obtained with two diffusion-weighted echo planar imaging (EPI DWI) scans with reversed phase-encoding polarities (each with multi-shell diffusion scheme with four different b-values and 104 directions; multi-band readout, voxel size of (1.5 × 1.5 × 1.5) mm^3^, TA = 6 min each, FOV = LR: 210 mm, PA: 210 mm, IS: 144 mm. In addition, fluid-attenuated inversion recovery (FLAIR) imaging was applied (TR = 10,000 ms; TE = 95 ms; TI = 2,500 ms; voxel size = (0.9 × 0.9 × 3.0) mm^3^, TA = 4 min 22 s, FOV = LR: 180 mm, PA: 240 mm, IS: 132 mm) for lesion identification and segmentation.

DWI data were pre-processed using FSL (topup and eddy_openmp (Andersson et al., 2003, Andersson and Sotiropoulos, 2016)). Fractional anisotropy (FA) and mean diffusivity (MD) were determined by fitting the data to the diffusion tensor model (Mrtrix3 (Tournier et al., 2019)), while the fibre-to-field angle θ was determined by constrained spherical deconvolution from the first peak of the orientation distribution function (ODF) (Tournier, Calamante, and Connelly 2007). The ratio between the amplitudes of the second to the first peak of ODF was calculated and used as a complementary measure to FA for WM anisotropy (further referred to as peak quotient, PQ). FA, PQ, and θ maps were transformed into the space of the GRE acquisitions by registering MD maps to the first echo of the T1w GRE sequence using Aladin from NiftyReg (Ourselin et al., 2001).

The white matter mask was obtained by combining Freesurfer segmentation (Desikan et al., 2006) of a synthetic T1w contrast (generated from the two GRE images using mri_synthesize, TR = 20 ms, FA = 30°, TE = 5 ms) with the five-tissue-type segmentation from Mrtrix3 (Smith et al., 2012) followed by binary erosion with a sphere (r = 2 mm). Voxels with crossing fibres were excluded from the WM mask based on FA < 0.6 and PQ > 0.3. In MS patients, lesion masks were obtained using nicMSlesions (Valverde et al., 2019) from FLAIR and T1w images (both images were registered to MNI space for each subject; the network was trained *de novo* on 220 MS subjects with semi-automatically generated labels). The obtained lesion masks were additionally dilated (r = 1 mm) to minimise possible segmentation or alignment issues and removed from the patients’ WM masks (the resulting mask is hereafter referred to as the normal appearing white matter (NAWM) mask).

**Fig. 1:**
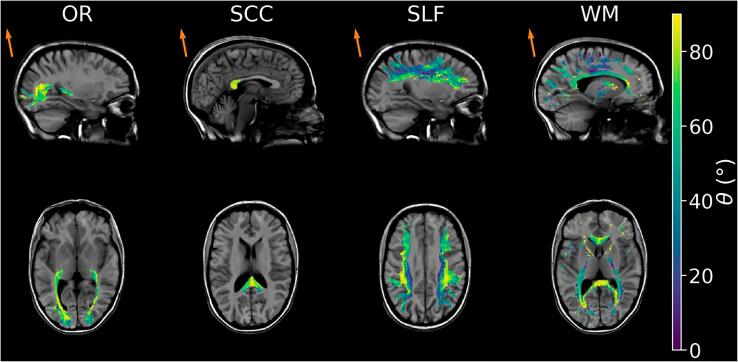
Distributions of fibre-to-field angles θ in a healthy volunteer for both the selected three white matter ROIs (eroded) and the WM mask (with crossing fibres excluded). The anatomical reference is provided by the synthetic T1w contrast. Each subfigure represents an average over a slab of 8 mm thickness. The orange arrows indicate the direction of ${\;\vec{B}}_{0}$.

Within such (NA)WM masks, the following three tract-based regions-of-interest (ROI) were selected: optic radiation (OR), splenium of corpus callosum (SCC), and superior longitudinal fascicles (SLF). Optic radiation and SLF were determined directly from the diffusion data using Tractseg (Wasserthal et al., 2018), and the three components of SLF were combined (Wang et al., 2016). The splenium of the corpus callosum was defined as the intersection of the dilated Freesurfer label for the posterior corpus callosum and the commissural fibres segmented by Tractseg (see appendix B in Sibgatulin et al., 2021 for details). Fig. 1 illustrates the choice of the ROIs together with the corresponding fibre-to-field distributions in a single subject.

PDw GRE images were rigidly registered to the T1w GRE images using Aladin (Ourselin et al., 2001). The relative frequency differences were obtained from the unwrapped phase data (3D path-following algorithm (Herráez et al., 2002) from scikit-image (version 0.16.1; Van der Walt et al., 2014)) of the PDw GRE scan, which were appropriately scaled and averaged over the last three echoes. Background frequency contributions were removed using V-SHARP (Schweser et al., 2011, Wu et al., 2012), and dipole inversion was performed using iLSQR (STI Suite; W. Li et al. (2015)). The resulting AMS values were referenced to the cerebrospinal fluid in the lateral ventricles. Phase data processing is described in more detail in Sibgatulin et al. (2021).

### 2.3 Data analysis

**Fig. 2:**
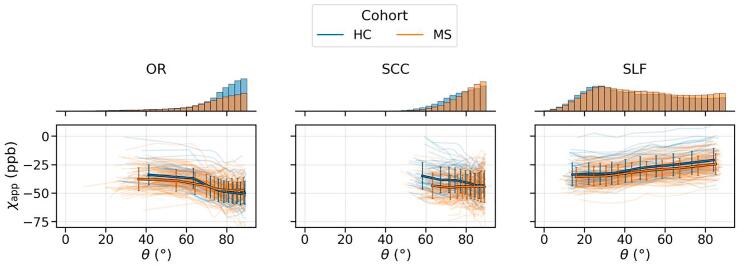
Orientation dependence of apparent magnetic susceptibility in different WM ROIs for the two analysed cohorts. Thin transparent lines correspond to individual subjects, while the thick lines with the error bars indicate the averaged trends with two standard deviations. The histograms represent the distributions of θ in each ROI, averaged across the subject in the respective group (scaled consistently across the subfigures).

For each subject, values of AMS and fibre-to-field angle θ were extracted from each of the three tracts (limited to the (NA)WM mask and with crossing fibres excluded as indicated above). For visualisation, data from each ROI were binned independently into 10 equally populated bins based on the deciles of θ for each subject, yielding an average AMS(θ) curve per ROI per subject (Fig. 2).

Estimation of δχ and $\chi_{iso}$, comparison of the two cohorts, and correction for a number of covariates were combined in a single Bayesian multilevel linear model (applied to each ROI independently). The model’s complete specification is given in the appendix (Equations A1–12), while Equation (3) summarises its structure:

(3) $$\chi_{app}^{voxel}=\chi_{iso}^{subj}+\beta_{iso}{AO}^{subj}+{\tau_{iso}^{coh}DD}^{subj}+{\lambda_{iso}^{coh}LL}^{subj}+\left({\delta \chi }^{subj}+\beta_{\delta \chi }{AO}^{subj}+{\tau_{\delta \chi }^{coh}DD}^{subj}+{\lambda_{\delta \chi }^{coh}LL}^{subj}\right)\cos^{2}\theta^{voxel},$$

where superscripts indicate the level of the respective variable. Importantly, both δχ and $\chi_{iso}$ (as they were defined in Equation (2)) were assumed to hold identical linear relationships with age (at onset) (AO), disease duration (DD), and lesion load (LL). Their age (at onset) dependence $\beta_{iso/\delta \chi }$ was assumed to be identical for both cohorts, while the dependences on disease duration and lesion load were defined within cohorts separately (thus superscript *coh*). Finally, $\chi_{iso}^{subj}$ and ${\delta \chi }^{subj}$ represent subject-specific isotropic AMS and apparent MSA adjusted for age, disease duration, and lesion load of the subject. As specified in Equations A5–6, these subject-specific parameters were modelled as samples from cohort-specific normal distributions defined by the respective means ($\mu_{iso/\delta \chi }^{coh}$) and standard deviations ($\sigma_{iso/\delta \chi }^{coh}$). Note that the dependencies on disease duration and lesion load in the HC cohort are nominal, as these values are set to 0 for each HC by definition. Due to this, $\tau_{iso/\delta \chi }^{HC}$ and $\lambda_{iso/\delta \chi }^{HC}$ are not reported for clarity.

This model ascribed the effect of healthy ageing to the subjects’ age at examination in the HC cohort and the age at onset in the MS cohort, hereafter referred to as age (at onset) and considered a single predictor. It was referenced to 30 years (median age of the HC cohort) and scaled by 10 years (standard deviation of age of the HCs). Disease duration was also measured in decades and set to 0 for HCs by definition. Lesion load was defined as the total volume of the lesions in a given ROI relative to the volume of the ROI (see Supplementary Figure S1 for the distributions). Before fitting, $\chi_{app}$ was standardised (demeaned and scaled by the standard deviation) across all subjects within each ROI independently. The model coefficients reported below were rescaled back to ppm. All described data scaling was performed to improve the performance of the employed inference algorithm.

The model was implemented in numpyro (0.6.0, Bingham et al., 2019, Phan et al., 2019) and the joint posterior distribution of the model parameters was sampled using Markov chain Monte Carlo (MCMC). Having the posterior represented by its samples allowed us to calculate the distribution of the effect size, η, defined as.

(4) $$\eta =\frac{\left(\mu_{iso/\delta \chi }^{MS}-\mu_{iso/\delta \chi }^{HC}\right)}{\sqrt{{\left[{(\sigma }_{iso/\delta \chi }^{MS\;}\right)}^{2}+{{(\sigma }_{iso/\delta \chi }^{HC\;})}^{2}]/2}}$$

This marginal posterior distribution and its 95% highest density interval (HDI) were reported as a mean to assess the significance of the difference between the two cohorts (Kruschke 2013).

## 3 Results

### 3.1 Orientation dependence of apparent magnetic susceptibility

Fig. 2 shows the dependence of $\chi_{\text{app}}$ on fibre-to-field angle θ in the regions-of-interest considered (along with the average distributions of θ). Notably, the distribution of the fibre-to-field angles in OR and especially SCC is very limited, as expected, covering just one-third of the entire angular range. Moreover, an average MS patient tends to have noticeably fewer voxels available in the OR due to the common localisation of lesions in this tract.

### 3.2 Comparison of isotropic AMS and apparent MSA across two cohorts

Fitting the model from Equation (3) (or more specifically Equations A1–12) results in a joint posterior distribution of three groups of parameters:

1. population level effects of age (at onset) ($\beta_{iso/\delta \chi }$), disease duration ($\tau_{iso/\delta \chi }^{MS}$), and lesion load ($\lambda_{iso/\delta \chi }^{MS}$);
2. $\chi_{iso}^{subj}$ and ${\delta \chi }^{subj}$, capturing individual variations that could not be explained by the aforementioned effects of age or disease;
3. the means ($\mu_{iso/\delta \chi }^{coh}$) and the standard deviations ($\sigma_{iso/\delta \chi }^{coh}$) summarizing the distribution of $\chi_{iso}^{subj}$ and ${\delta \chi }^{subj}$.

**Fig. 3:**
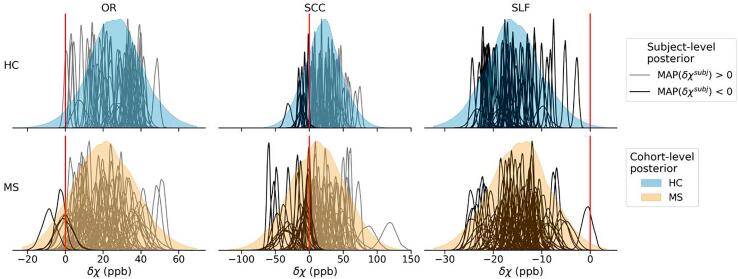
Marginal posterior distributions of ${\delta \chi }^{subj}$ (in black or grey) for the healthy controls (top row) and the MS patients (bottom row). Subjects represented by black lines are characterised by a negative maximum a posteriori (MAP) estimate. Sampled cohort-level distributions are shown in colour. The red vertical lines indicate δχ = 0 ppb. The scale of the x-axis is shared across the cohorts, but not across the ROIs.

**Fig. 4:**
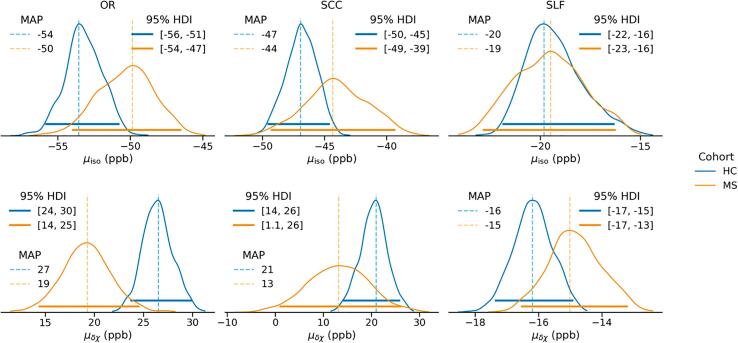
Marginal posterior distributions of the cohort means $\mu_{\text{iso}}^{coh}$ (top row) and $\mu_{\delta \chi }^{coh}$ (bottom row). Dashed vertical lines represent the maximum a posteriori estimate, while solid horizontal lines indicate the 95% HDI (i.e., credible interval of the estimated mean, and not a measure of the cohort-level distribution spread).

We start with the second group of parameters to illustrate the relation between the individual ${\delta \chi }^{subj}$ estimates and the inferred cohort-level distributions. Fig. 3 presents the marginal posterior probability distributions of ${\delta \chi }^{subj}$ for subjects grouped in their respective cohort (per row). Recall that the model in Equation (3) assumed that the ${\delta \chi }^{subj}$ values were drawn from cohort-level normal distributions, described by $\mu_{iso/\delta \chi }^{coh}$ and $\sigma_{iso/\delta \chi }^{coh}$ (cf. Equations A5–6). The marginal posterior distributions of $\mu_{iso/\delta \chi }^{coh}$ are shown and discussed further in Fig. 4, while samples from the joint posterior were used to generate a number of cohort-level distributions shown in Fig. 3 (filled). An equivalent representation of the marginal posterior for $\chi_{iso}^{subj}$ is shown in Supplementary Figure S2.

As mentioned above, previous research strongly suggests that δχ of white matter fibres is *positive* (notably, this prior knowledge was not included in the priors of the model, cf. Equation A10). In the light of this, Fig. 3 highlights individual subjects with negative maximum a posteriori (MAP) ${\delta \chi }^{subj}$ estimate (all subjects in the SLF case). While ${\delta \chi }^{subj}$ has a consistent sign in OR and SLF for almost all subjects, its estimates appear much more scattered in the splenium, especially in the MS cohort (note the axis spanning three times the range of values observed in OR). This dispersion of the cohort-level distributions is captured by their respective standard deviations, $\sigma_{iso/\delta \chi }^{coh},$ shown in Supplementary Figure S3. Their respective means $\mu_{iso/\delta \chi }^{coh},$ which allow comparison of the two cohorts, are shown in Fig. 4 (and are also reported in Supplementary Table S1).

Note that Fig. 4, along with the subsequent figures, reports marginal posterior distributions of the estimated parameters, together with their MAP estimates and the 95% Highest Density Interval (HDI), the narrowest range of values containing 95% of the probability mass. The latter can be seen as the Bayesian counterpart of the 95% confidence interval, and its position with respect to zero is considered as evidence for or against the effect in question.

The means reported in Fig. 4 can be viewed as expected values of $\chi_{iso}$ and δχ for healthy and MS subjects aged 30 years old (in addition, in the case of the MS cohort, at disease onset and without lesions in the corresponding ROI). For both $\chi_{iso}$ and δχ the estimated means tend to agree in SLF, while showing consistent differences between the cohorts in OR and SCC.

**Fig. 5:**
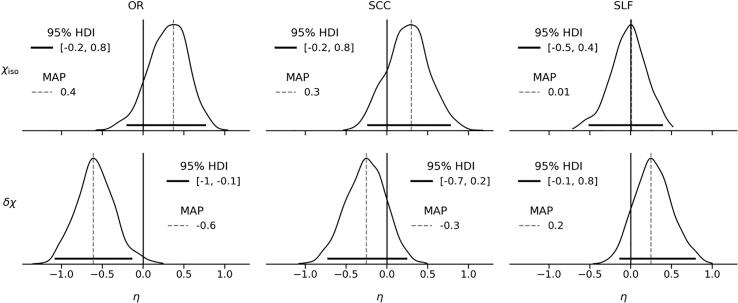
Marginal posterior distributions of the effect sizes. Dashed vertical lines represent the MAP estimate, while solid horizontal lines indicate the 95% HDI. The solid vertical lines show the location of $\;\mu_{iso/\delta \chi }^{MS\;}-\;\mu_{iso/\delta \chi }^{HC\;}=\;0.$ All subfigures share the same x-axis scale.

Given that the posterior is a joint distribution of all parameters from Equations A1–12, and each sample from the posterior includes a combination of $\mu_{iso/\delta \chi }^{coh}$ and $\sigma_{iso/\delta \chi }^{coh}$ consistent with the data, the effect size can be conveniently estimated using Equation (4) (shown in Fig. 5). Using this definition, we find that only δχ in OR can be confidently considered different between the two cohorts (with the MAP effect size of -0.6 and only 1.2% of marginal posterior probability above 0). Besides, both $\chi_{iso}$ and δχ show the same trends of change between the cohorts in OR and SCC, whereas δχ in SLF shows the opposite sign of the effect ($\mu_{\delta \chi }^{MS\;}>\mu_{\delta \chi }^{HC\;}$) compared with OR and SCC.

### 3.3 Effects of age, disease duration, and lesion load

As mentioned earlier, to separate the effects of healthy ageing and disease duration in the MS cohort, we included two predictors in the linear model from Equation (3) (age at disease onset and disease duration) and assumed that the effect of age at onset is comparable to that of healthy ageing in the control cohort (represented by $\beta_{iso}$ and $\beta_{\delta \chi }$).

**Fig. 6:**
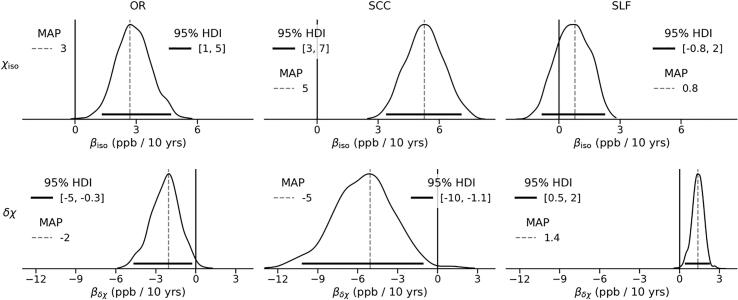
Marginal posterior distributions of the effects of healthy ageing, captured by $\beta_{\text{iso}}$ (top row) and $\beta_{\delta \chi }$ (bottom row). Dashed vertical lines represent the MAP estimate, while solid horizontal lines indicate the 95% HDI. Solid vertical lines highlight the location of $\beta_{\text{iso}/\delta \chi }=0.$ The scale of the x-axis is shared within each row.

Fig. 6 shows the marginal posterior distributions of such effect in each ROI. Both $\chi_{iso}$ and δχ showed a significant response to age (in the sense of the 95% HDI excluding the 0) in almost every ROI. In particular, $\chi_{iso}$ increases with age, showing a significant increase in the OR and SCC (by 1–5 and 3–7 ppb / 10 years, respectively) and a trend toward an increase in SLF (78% of the probability mass is above $\beta_{iso}=0$). In contrast, δχ decreases significantly with age in OR (by 0–5 ppb / 10 years) and to a greater extent in SCC (by 1.1–10 ppb / 10 years), whereas it increases significantly in SLF (albeit only by 0.5–2 ppb / 10 years).

**Fig. 7:**
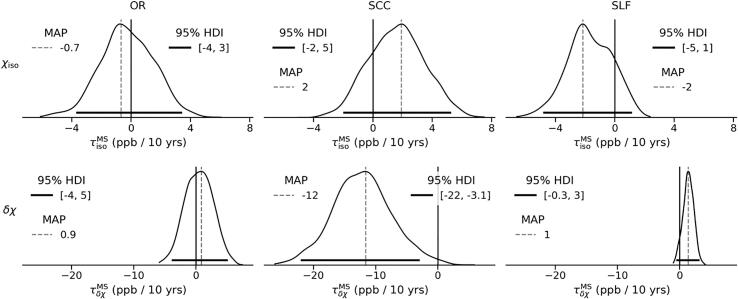
Marginal posterior distributions of disease duration, captured by $\tau_{\text{iso}}^{\text{MS}}$ (top row) and $\tau_{\delta \chi }^{\text{MS}}$ (bottom row). Dashed vertical lines represent the MAP estimate, while solid horizontal lines indicate the 95% HDI. Solid vertical lines highlight the location of $\tau_{\text{iso}/\delta \chi }^{\text{MS}}\;=\;0$. The scale of the x-axis is shared within each row.

Complementary to the effect of healthy ageing, represented by $\beta_{iso/\delta \chi }$, the effect of disease duration is captured by $\tau_{iso/\delta \chi }^{MS}$, shown in Fig. 7. Among all ROIs, only SCC shows a significant decrease in δχ with disease duration (although the 95% HDI spans a wide range between 3 and 22 ppb). At the same time, SLF shows the opposite pattern of response to disease duration, whereas OR shows virtually no response at all.

**Fig. 8:**
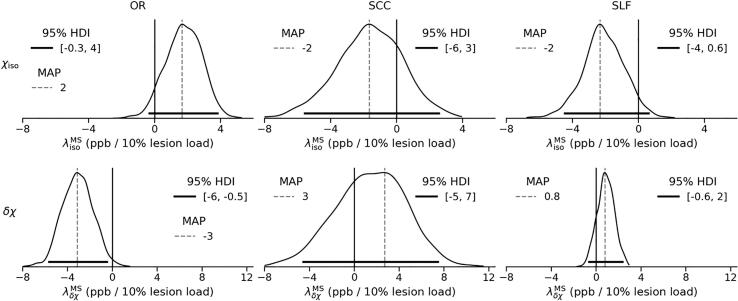
Marginal posterior distributions of the effect of the lesion load in each ROI, represented with of $\lambda_{\text{iso}}^{\text{MS}}$ (top row) and $\lambda_{\delta \chi }^{\text{MS}}$ (bottom row). Dashed vertical lines represent the MAP estimate, while solid horizontal lines indicate the 95% HDI. Solid vertical lines highlight the location of $\lambda_{\text{iso}/\delta \chi }^{\text{MS}}\;=\;0$. The scale of the x-axis is shared within each row.

Finally, Fig. 8 shows the effect of lesion load. Most notably, in OR, δχ decreases significantly in the presence of lesions by 0.5–6 ppb / 10 % lesion load, whereas $\chi_{iso}$ tends to increase by 0–4 ppb / 10 % lesion load. The effect of the lesion load in SCC and SLF, however, shows the opposite trend for both $\chi_{iso}$ and δχ (more pronounced in SLF).

The significant effects reported above are summarized in Supplementary Table S2.

## 4 Discussion

In this study, we examined apparent magnetic susceptibility in three white matter fibre tracts in healthy volunteers and MS patients to explore the potential of the apparent magnetic susceptibility anisotropy, δχ, for indicating demyelination in normal-appearing white matter.

It is generally accepted that the apparent magnetic susceptibility in grey matter is primarily determined by the presence of tissue iron (Langkammer et al., 2012, Deistung et al., 2013), whereas the interpretation of QSM values in white matter is more challenging due to the opposing susceptibility effects of iron and myelin sheaths (Liu et al., 2011, Schweser et al., 2011, Lee et al., 2012).

Our hypothesis underlying the study was that the apparent magnetic susceptibility anisotropy, δχ, is sensitive exclusively to the integrity and amount of myelin present. Such exclusive sensitivity cannot be attributed to the isotropic AMS, $\chi_{iso}$, because it includes the effects of iron as well as any residual — but orientation-independent — contributions from myelin sheaths in addition to other tissue components.

Importantly for the following discussion and in line with the interpretation of magnetic susceptibility anisotropy by Li et al. (2012a), the apparent MSA should be positive, with lower values indicating stronger myelin degradation. This is also consistent with previously published results (Lee et al., 2010, Wharton and Bowtell, 2012, Wharton and Bowtell, 2015, Li et al., 2012a, Li et al., 2012b). From this perspective, negative MSA values would indicate a significant change in the susceptibility tensor of myelin lipids, which does not seem readily plausible. Negative MSA estimates, however, have been reported for both tensor (Li et al., 2012b, Wisnieff et al., 2013) and apparent MSA (Xiao et al., 2014, Lancione et al., 2017, Sibgatulin et al., 2021) and were considered artefactual (Li et al., 2012b, Wisnieff et al., 2013, Sibgatulin et al., 2021). We discuss the possible origin of this observation below.

### 4.1 Range of estimated $\chi_{iso}$ and δχ values

The distribution of apparent MSA estimated in individual subjects (${\delta \chi }^{subj}$) varies qualitatively among the three ROIs but appears to be consistent across both cohorts (see Fig. 3). In both cohorts, the apparent MSA is predominantly positive in OR and negative in SLF, while it covers the largest range of values in SCC. Given the presumed role of MSA, the negative estimates of apparent MSA do not appear to be biophysically justified, but even in SCC, they cannot be dismissed as outliers, as is evident from their distributions in Fig. 3. Such distributions may indicate systematic errors in the estimation of δχ and must be considered when interpreting the differences between the two cohorts.

As for the SLF, not a single subject in either cohort showed a positive apparent MSA, suggesting that δχ may be systematically misestimated throughout the entire ROI. Nevertheless, we included this ROI in our analysis for completeness due to its prominent size as well as the observed robustness of the results.

The marginal posterior distributions of the age-corrected mean apparent MSA in the HC cohort ($\mu_{\delta \chi }^{HC}$) in OR (MAP = 27 ppb, 95% HDI = 24–309 ppb) and SCC (MAP = 21 ppb, 95% HDI = 14–26 ppb) appear comparable to, but slightly larger than, previously published estimates of MSA (6–12 ppb in the human corpus callosum *ex vivo* at 7 T (Lee et al., 2010), 22 ppb in human WM *in vivo* at 3 T (Li et al., 2012a), 16 ppb in human WM *in vivo* at 7 T (Wharton and Bowtell 2012), 5 and 10 ppb in human posterior thalamic radiation and SCC respectively, measured at 7 T *in vivo* (Li et al., 2012b), or 17 ppb in porcine optic nerve at 7 T (Wharton and Bowtell 2015)) as well as apparent MSA (10 ppb across multiple WM tracts measured *in vivo* (Xiao et al., 2014) or 14 ppb and 21 ppb in corticospinal tract of paediatric cerebral palsy patients (Zhang et al., 2021) measured at 3 T). Such differences likely result from discrepancies in the processing pipeline, such as the extent of background field removal, but such discrepancies should affect the scale of AMS globally and affect all ROIs identically.

Direct comparison of the isotropic AMS with previously published bulk magnetic susceptibility values is generally not straightforward. However, based on the distributions of fibre-to-field angles in OR and SCC, $\chi_{iso}$ tends to agree well with the mean AMS in the corresponding ROI. For SLF, with its relatively homogeneous distribution of θ, the mean AMS is expected to be comparable to $\chi_{iso}+\delta \chi /2\approx -30$ ppb. All values appear to be in the correct order of magnitude when compared with previously reported white matter susceptibility values. In addition, Li et al. (2012b) reported mean magnetic susceptibilities (estimated from a tensor reconstruction of multiple head orientation acquisitions) of posterior thalamic radiation < SCC < corona radiata (anterior and superior), which is in good agreement with the observed ranking of $\chi_{iso}$ (OR < SCC < SLF, see Fig. 4).

### 4.2 Limitations of the approach to estimating apparent MSA

Before turning to a comparison of the results obtained in the two cohorts, we briefly discuss the assumptions made in the present analysis and the corresponding limitations.

The approach used in this paper (as well as in Sibgatulin et al., 2021) is based on the relation between the apparent magnetic susceptibility and the underlying tensor MSA considered by Li et al. (2012a). This model: 1) assumes that the field perturbations produced by lipid molecules in the myelin sheath can be consistently described as a convolution of a scalar apparent susceptibility (AMS) with the dipole kernel used in QSM and 2) implies that such an AMS captures the anisotropic effect in its orientation dependence described in Equation (1). This model is attractive because of its simplicity and has been referred to in a number of papers (Lee et al., 2011, Lancione et al., 2017, Zhang et al., 2021, Sibgatulin et al., 2021), but its applicability is not yet fully clear. Thus, the present work should be viewed as an attempt to empirically find out where limitations of the model are, while a better theoretical understanding of these limitations is still needed.

#### 4.2.1 Susceptibility reconstruction

First and foremost, the most challenging aspect of quantitative susceptibility mapping as well as susceptibility tensor reconstruction is the inherently ill-posed nature of the field-to-source inversion step. For any single acquisition, the components of the susceptibility tensor, which are perpendicular to the given ${\vec{B}}_{0}$ direction do not contribute to the observed field variation (Liu 2010) and undergo a rather ill-conditioned inversion process (Wisnieff et al., 2013). In any of the proposed tensor reconstruction approaches (Liu, 2010, Li et al., 2012b, Wisnieff et al., 2013) both issues are addressed by acquiring and combining phase information at different sample orientations with respect to ${\vec{B}}_{0}$.

Wisnieff et al. (2013) simulated the error propagation in the reconstruction of a cylindrically symmetric tensor with 2 components, and showed that 3 uniformly distributed sampling angles (i.e., angles defining the orientation of the object with respect to ${\vec{B}}_{0}$) yield an accurate reconstruction of tensor-based MSA. However, the set of anatomically possible head orientations covers only a relatively narrow range of sampling angles, leading to substantial misestimates of different tensor components (and thus MSA) in different fibre orientations (underestimation of MSA in orientations parallel to ${\vec{B}}_{0}$ and overestimation in perpendicular orientations). It is important to emphasize that the ill-conditioned nature of reconstructing the susceptibility tensor from a finite set of sample orientations has been shown to lead to systematic θ-dependent biases in the estimated tensor components (Li et al., 2012b, Wisnieff et al., 2013). It is thus reasonable to assume that any contribution of the two tensor components ($\chi_{\|}$ and $\chi_{⊥}$) to AMS is subject to similar θ-dependent errors. Although the scalar QSM reconstruction makes such a connection much less transparent, it is conceivable that the estimation of apparent MSA, performed across a range of fibre-to-field angles, can result in complex biases in δχ. To our knowledge, no study has yet been conducted on error propagation in the estimation of apparent MSA, which would be most valuable for the interpretation of the observed results. It would be especially interesting with regard to the negative δχ observed in SLF, as in an experiment with three head orientations, Wisnieff et al. reported negative tensor MSA results: a fraction of estimates in SCC and all estimates in the genu of the corpus callosum (GCC), while all ROIs showed positive values when more orientations were used.

This possible relation to the results from the studies addressing the susceptibility tensor points to a rather fundamental issue with the inverse problem, potentially shared by different inversion algorithms. Although comparison of different approaches to QSM was not a major part of this study, we have additionally considered the effect of substituting iLSQR for rapid two-step dipole inversion (RTS; Kames et al., 2018). The general pattern of the AMS orientation dependence remains unchanged as indicated in Supplementary Figure S4 by the trend averaged across the cohort of healthy subjects.

#### 4.2.2 Aggregating across ROIs

Additional limitations may arise from the approach that aggregates AMS and fibre-to-field angle θ over the extent of a given WM track. In particular, this approach assumes that AMS does not exhibit spurious variations over the ROI under consideration. This assumption may not hold for WM areas bordering boundaries with strong susceptibility variations (e.g., the internal capsule, which runs near the strongly paramagnetic globus pallidus, or the GCC, which lies near the lateral ventricles and frontal sinuses (Wisnieff et al., 2013)). Given the variability in head orientation and individual anatomy, confounding AMS distributions with structured artefacts caused by imperfect dipole inversion may be a plausible source of random variation in the AMS orientation dependence (and consequently in the estimated ${\delta \chi }^{subj}$) in the SCC. However, this perspective does not provide a satisfactory explanation for the very robust negative δχ in SLF — a large anatomical region that appears to be relatively distant from sources of strong field variations.

Besides said spurious variation of AMS, genuine variation in the underlying tensor MSA across the ROIs may as well confound the proposed estimation of apparent MSA, especially if both MSA and fibre-to-field angle are correlated in a given ROI. This constitutes a fundamental limitation of the approach and turns the estimated apparent MSA, loosely speaking, into a kind of weighed average for the underlying MSA. Nevertheless, we find it unlikely that aggregation over a genuine (e.g. positive) MSA distribution can lead to a qualitatively inaccurate (e.g. negative) aMSA.

#### 4.2.3 Reliance on the diffusion data

Besides the many pitfalls of susceptibility reconstruction, additional sources of potential errors may lie in the calculation of the fibre-to-field angle maps, as the latter are obtained from a complex processing of diffusion MR data. It should be pointed out that in this study, as in many other diffusion-guided approaches (Li et al., 2012b, Wisnieff et al., 2013, Bao et al., 2021), fibre orientation was considered error-free, which cannot be assumed universally. However, for the high FA voxels included in the analysis, this seems to be an acceptable assumption (see Methods 2.2).

#### 4.2.4 Myelin contribution in $\chi_{iso}$

Finally, while δχ is thought to be primarily related to the amount and state of myelin, the effect of the latter on the isotropic AMS, $\chi_{iso}$, cannot be disentangled from a paramagnetic contribution of tissue iron without considering additional parameters. Myelin water fraction has been shown to be a useful proxy for myelin concentration in models of orientation dependence of $R_{2}^{*}$ (Lee et al., 2017) and could be a valuable addition to the outlined approach. Alternatively, $R_{2}^{\prime }$ was recently used as an independent measure of the local field dispersion to separate positive and negative contributions to AMS (Shin et al., 2021).

#### 4.2.5 Anisotropic cerebral vasculature

Another potential source of magnetic field perturbation that has not been considered in this work, is the venous blood vessels. Such a contribution is generally neglected due to the small volume fraction of the vascular network. With a number of studies suggesting that blood vessels in WM are predominantly parallel to the WM tracts (Nonaka et al., 2003, Hernández-Torres et al., 2017), and the fact that such contributions depend on the angle between the vessel and the main magnetic field (Sedlacik et al., 2007), we point to the possibility that the venous network may influence the extracted apparent magnetic susceptibility anisotropy in a WM ROI. However, additional research in this direction is certainly needed, and currently we do not have a viable modelling approach as to whether the addition of a venous contribution to susceptibility, for example, could at least partially explain the unexpected observation in the SLF.

### 4.3 Effects of ageing and disease progression

The isotropic AMS shows a significant increase with healthy ageing in both OR and SCC. This increase is consistent with previously published findings of an age-dependent increase in magnetic susceptibility in adult white matter (W. Li et al., 2014). Furthermore, the rate of change of $\chi_{iso}$ in HC, ranging from a statistically non-significant 0.8 ppb/10 year in SLF to 3 ppb/10 year in OR to 5 ppb/10 year in SCC, appears to be of the same order of magnitude as the linear increase of AMS in adults (1–3 ppb/10 year in SCC and OR) reported by W. Li et al. (2014). The authors associated such an increase in AMS with age with demyelination, which inevitably contributes to both δχ and $\chi_{iso}$. It should, however, be noted that the isotropic part of AMS is potentially affected by a number of factors that occur together in normal ageing, such as myelin degradation and increases in tissue iron concentration. Both factors lead to an increase in isotropic AMS, which seems to be consistent with the observed behaviour of $\chi_{iso}$ in OR and SCC. Furthermore, Hametner et al. (2013) reported a decrease in age-corrected iron concentration in NAWM in chronic MS. Although this change in iron concentration cannot be directly compared to the change in $\chi_{iso}$ with disease duration (i.e., $\tau_{iso}^{MS}$) due to the expected concomitant decrease in the myelin concentration, it may be associated with the observed weaker response of $\chi_{iso}$ to disease duration in OR and SCC, as well as the decreasing trend in SLF (i.e., in all ROIs $\tau_{iso}^{MS}<\beta_{iso}$).

Comparison of the apparent magnetic susceptibility anisotropy (δχ) across the two cohorts (via $\mu_{\delta \chi }^{MS}-\mu_{\delta \chi }^{HC}$ and the respective effect size) shows a significant decrease in AMS in OR of patients with RRMS (effect size of -0.6) and such a trend in SCC. Furthermore, δχ decreases significantly with age (at onset) in both ROIs, additionally with disease duration in SCC, and with increasing lesion load in OR. Given the hypothesized relationship between δχ and myelin (Li et al., 2012a, Zhang et al., 2021), it is conceivable that the observed decrease in apparent MSA — whether with healthy ageing or disease progression — is associated with deterioration of myelin sheaths in NAWM, at least in OR. Specifically, the observed strong δχ response to lesion load in OR may echo previously reported axonal damage and demyelination in perilesional NAWM (Singh et al., 2017).

Interestingly, in the cohort of MS patients studied, OR appears to be most strongly affected by lesions (as measured by the ROI fraction, see Supplementary Figure S1), which may explain the observed sensitivity of apparent MSA in OR to the effect of lesion load, especially when compared with the other ROIs.

This result appears to be consistent with findings of Yu et al. (Yu et al., 2019), who studied a cohort of RRMS patients and reported significantly increased radial diffusivity (RD) and decreased FA in the forceps major, which includes the SCC and is directly adjacent to the optic radiation. The authors interpreted these findings as indicative of demyelination (for which increased RD is considered a particularly specific predictor (Bennett et al., 2010)) and axonal loss. Consistent, moreover, is the fact that neither Yu et al. nor our study found a statistically significant difference between the cohorts with AMS or isotropic AMS ($\mu_{iso}^{coh})$, respectively. Yu et al. suggested that this discrepancy between RD and magnetic susceptibility might be explained by a concomitant decrease in tissue iron content that masks the effect of demyelination on AMS. Such a change in iron was not uniquely identified in either study but is consistent with previously reported results (Hametner et al., 2013).

In SCC, the observed difference in mean δχ (see Fig. 3, Fig. 4, Fig. 5) seems to be due to a substantial proportion of MS subjects showing strongly negative apparent MSA, which is problematic from a biophysical perspective, as discussed above. Similarly, the apparent MSA in SLF remains negative in both cohorts and is thus difficult to interpret biophysically. Interpretation of the trend observed in SLF toward a decrease in the absolute value of δχ in the MS cohort (effect size of 0.2) as well as with age and increased lesion load, would require a better understanding of the mechanisms determining the negative apparent MSA in SLF.

### 4.4 Related work and possible future directions

It is worth noting the relation of the proposed orientation-resolved approach to the comparison of AMS values across the two cohorts, as presented, e.g., by Yu et al. (2019). The latter, while much simpler to perform, aggregates AMS values across the range of fibre orientations found in a given tract-based ROI, but this may mask potential differences between the two cohorts. Hernández-Torres et al. (2015) reported an improvement in discrimination between MS patients and healthy controls by correcting $R_{2}^{*}$ for its orientation dependence. The authors argued that such an orientation dependence of the relaxation rate constant introduces innate dispersion of $R_{2}^{*}$ histograms, hiding small differences between the cohorts. We observed a similar effect when the statistical analysis presented in this paper was modified to exclude any consideration of orientation dependence (i.e., without the $b^{subj}\cos^{2}\left(\theta^{voxel}\right)$ term in Equation A2). The resulting age- and disease progression-corrected AMS values revealed an effect of the same sign as reported in Fig. 5 for $\chi_{iso}$, but of smaller magnitude, suggesting confounding by the effects of orientation dependence. This analysis was not included in the present manuscript in order to focus on the more physically meaningful representation of AMS by the components in Equation (2).

Interestingly, the orientation dependence of $R_{2}^{*}$ reported by Hernández-Torres et al. appeared to be reduced in the MS cohort, although no significant difference was observed between the groups (possibly due to the effect of crossing fibres, which were not excluded). In contrast, in the present study, it is the orientation dependence of AMS reflected in δχ that shows the most striking difference between the MS patients and the healthy subjects, while the orientation-independent component, $\chi_{iso}$, only suggests an increasing trend in the MS cohort. This observation is consistent with the hypothesis of concurrent iron and myelin loss, both of which would lead to a decrease in orientation-independent $R_{2}^{*}$, but would cancel out in their effect on $\chi_{iso}$. This suggests a potentially interesting future direction of incorporating the orientation dependence model from Equation (2) into an approach to separate AMS into positive and negative contributions (Shin et al., 2021).

Finally, as shown in Sibgatulin et al. (2021), the orientation dependence of AMS, observed in many other WM regions, does not appear to be consistent with Equation (2), leaving the question of the applicability of the proposed analysis in these ROIs open and largely dependent on further critique of the model in Equations (1), (2) (specifically, its completeness and applicability of single orientation QSM to be used as input for apparent MSA estimation). This question was explored in more detail in sections 4.1 and 4.6 of Sibgatulin et al. (2021).

## 5 Conclusion

In this study, we investigated changes in apparent magnetic susceptibility in NAWM in a cohort of RRMS patients considering susceptibility anisotropy and using clinically feasible single-orientation acquisitions. In the MS cohort, the apparent magnetic susceptibility anisotropy (δχ) showed a significant decrease in OR and a decreasing trend in SCC. Moreover, δχ in OR decreased with increasing lesion load in the ROI, whereas δχ in SCC decreased with disease progression.

The presented approach appears to be limited by the stability of δχ estimation in SCC and by the possibility of a systematic misestimation of AMS when using single-orientation acquisition (as reflected by a robustly negative sign of δχ in SLF). Despite the limitations of ROI-specific estimation, we consider our single-orientation approach to study degenerative changes in WM to be valuable and worthy of further investigation because of its clinical feasibility.

## CRediT authorship contribution statement

**Renat Sibgatulin:** Software, Formal analysis, Data curation, Investigation, Writing – original draft, Visualization. **Daniel Güllmar:** Conceptualization, Software, Data curation, Writing – review & editing. **Andreas Deistung:** Conceptualization, Writing – review & editing. **Christian Enzinger:** Resources. **Stefan Ropele:** Conceptualization, Data curation, Resources, Project administration, Funding acquisition, Writing – review & editing. **Jürgen R. Reichenbach:** Conceptualization, Resources, Project administration, Funding acquisition, Writing – review & editing.

## Declaration of Competing Interest

The authors declare that they have no known competing financial interests or personal relationships that could have appeared to influence the work reported in this paper.

## Appendix

### Complete specification of the Bayesian multilevel linear model used in the study

(A1) $$\chi_{\text{app}}^{\text{voxel}}\sim \;\text{Normal}\left(\mu^{\text{voxel}},\;\sigma^{\text{subj}}\right)$$

(A2) $$\mu^{\text{voxel}}=a^{\text{subj}}+b^{\text{subj}}\cdot \cos^{2}{(\theta }^{\text{voxel}})$$

(A3) $$a^{\text{subj}}=\chi_{\text{iso}}^{\text{subj}}+\beta_{\text{iso}}\cdot \text{A}{\text{O}}^{\text{subj}}+\tau_{\text{iso}}^{\text{coh}}\cdot \text{D}{\text{D}}^{\text{subj}}+\lambda_{\text{iso}}^{\text{coh}}\cdot \text{L}{\text{L}}^{\text{subj}}$$

(A4) $$b^{\text{subj}}=\delta \chi^{\text{subj}}+\beta_{\delta \chi }\cdot \text{A}{\text{O}}^{\text{subj}}+\tau_{\delta \chi }^{\text{coh}}\cdot \text{D}{\text{D}}^{\text{subj}}+\lambda_{\delta \chi }^{\text{coh}}\cdot \text{L}{\text{L}}^{\text{subj}}$$

(A5) $$\chi_{\text{iso}}^{\text{subj}}\sim \;\text{Normal}\left(\mu_{\text{iso}}^{\text{coh}},\;\sigma_{\text{iso}}^{\text{coh}}\right)$$

(A6) $$\delta \chi^{\text{subj}}\sim \;\text{Normal}\left(\mu_{\delta \chi }^{\text{coh}},\;\sigma_{\delta \chi }^{\text{coh}}\right)$$

(A7) $$\beta_{\mathrm{iso}/\delta \chi }\sim \text{Normal}\left(0,\;0.5\right)$$

(A8) $$\tau_{\text{iso}/\delta \chi }^{\text{coh}}\sim \;\text{Normal}\left(0,\;0.5\right)$$

(A9) $$\lambda_{\text{iso}/\delta \chi }^{\text{coh}}\sim \;\text{Normal}\left(0,\;1.5\right)$$

(A10) $$\mu_{\text{iso}/\delta \chi }^{\text{coh}}\sim \;\text{Normal}\left(0,\;1\right)$$

(A11) $$\sigma_{\text{iso}/\delta \chi }^{\text{coh}}\sim \;\text{HalfNormal}\left(2\right)$$

(A12) $$\sigma^{\text{subj}}\sim \;\text{HalfNormal}\left(1\right)$$

Equations A(1)–(4) represent the likelihood of the linear model and are summarised in Equation (3), whereas Equations A5–12 state the priors imposed on the model’s parameters. The priors in Equations A7–10 are weakly informative, i.e., they are defined by normal distributions with zero mean. Note that such weakly informative priors do not constrain the shape of the posterior distribution (or its marginals), and are chosen to indicate the target range of the parameter space, while still allowing the data and the likelihood term to drive the update of the posterior.

The use of the normal distribution for the likelihood in A1 reflects the assumption of the noise in the observations (i.e., $\chi_{app}^{voxel}$) following a Gaussian distribution with zero mean and standard deviation $\sigma^{subj}$, which itself is given a prior specified in A12. HalfNormal is the positive half of a normal distribution with zero mean and as such is characterised only by its standard deviation. The values for all standard deviations in the model were selected after a series of prior predictive simulations to ensure that the model captures the variance observed in the data (see Supplementary Figure S5 for an example of prior and posterior predictive distributions). Equations (A5 and A6) show centred parametrisation of hierarchical priors, which was automatically decentred (Gorinova et al., 2020).
