# Supplementary material for: Magnetic susceptibility anisotropy in normal appearing white matter in multiple sclerosis from single-orientation acquisition
Source: Neuroimage Clin. 2022 May 28;35:103059. doi: 10.1016/j.nicl.2022.103059 (PMC9163587; doi:10.1016/j.nicl.2022.103059)
Supplement: Supplementary data 1 [file mmc1.docx]

# Supplementary information


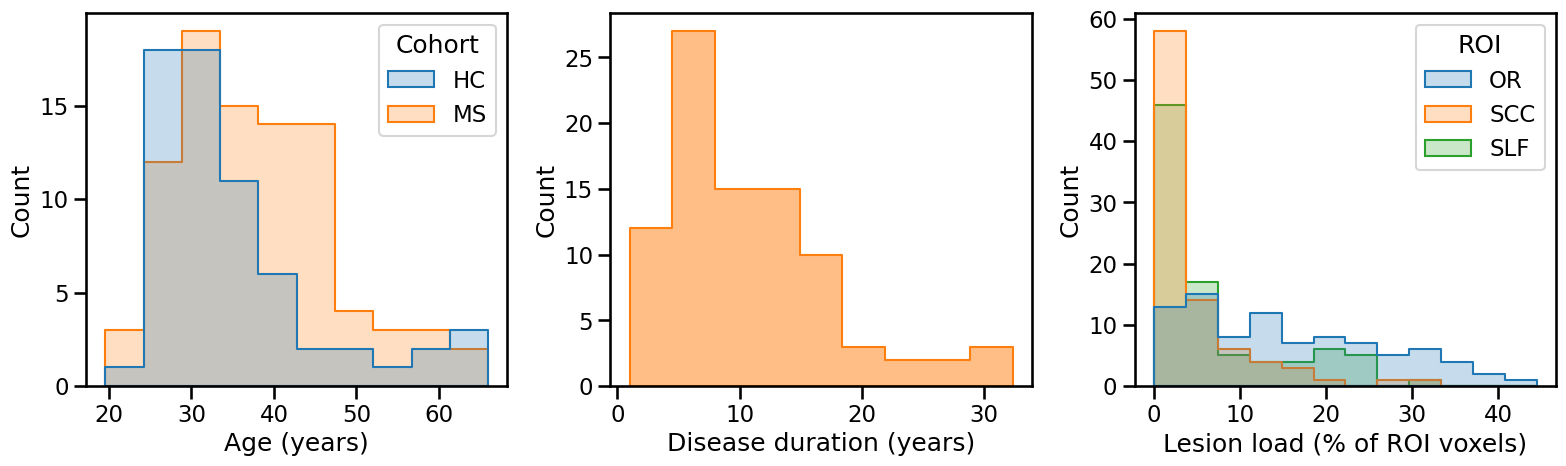


**Figure S1:** Distributions of demographic and clinical parameters in the included cohorts. Left: Age distribution in the two included cohorts. Centre: Distribution of disease duration among the patients. Right: Distribution of the fraction of the voxels in a given ROI that were attributed to lesions in each patient (one value per patient).

***Figure S2:*** *Marginal posterior distributions of* $\chi_{\text{iso}}^{\text{subj}}$ *(in black) for the healthy controls (top row) and the MS patients (bottom row). Cohort-level posteriors are shown in colour. The x-axis scale is the same for the two cohorts but not for the ROIs.*


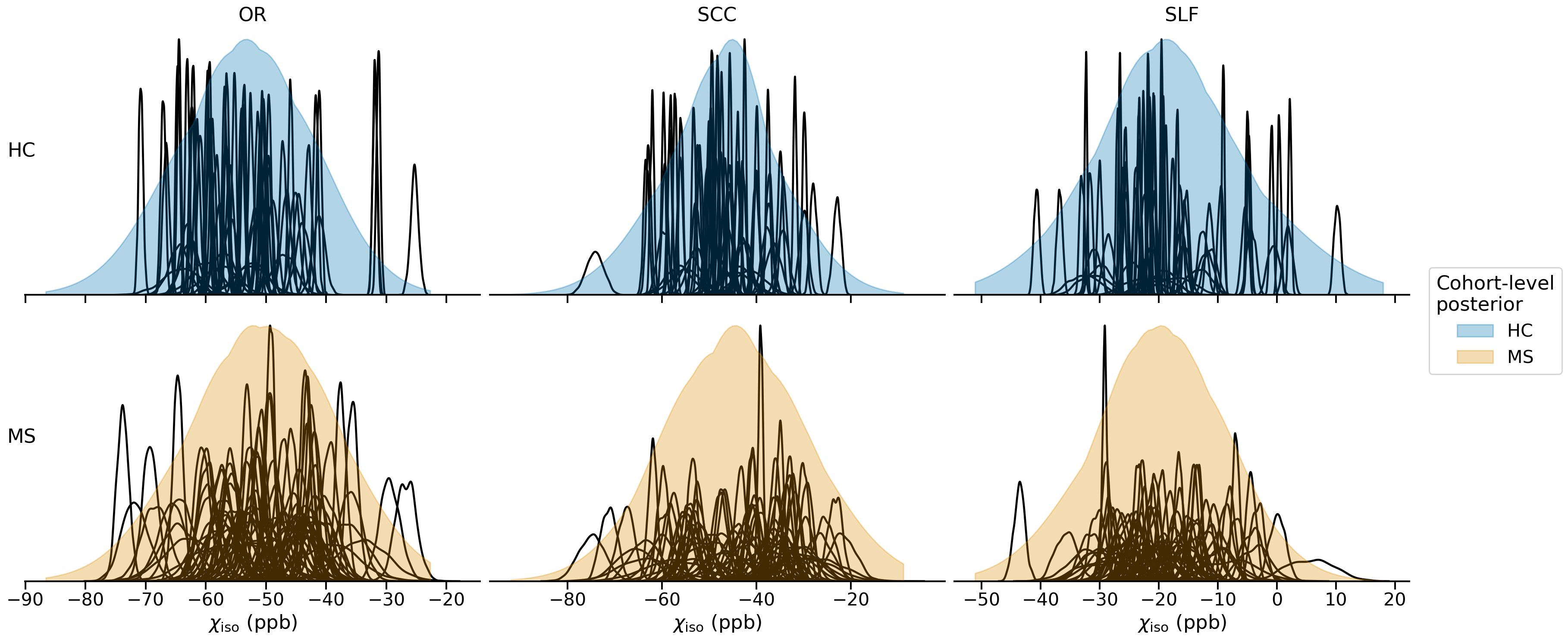

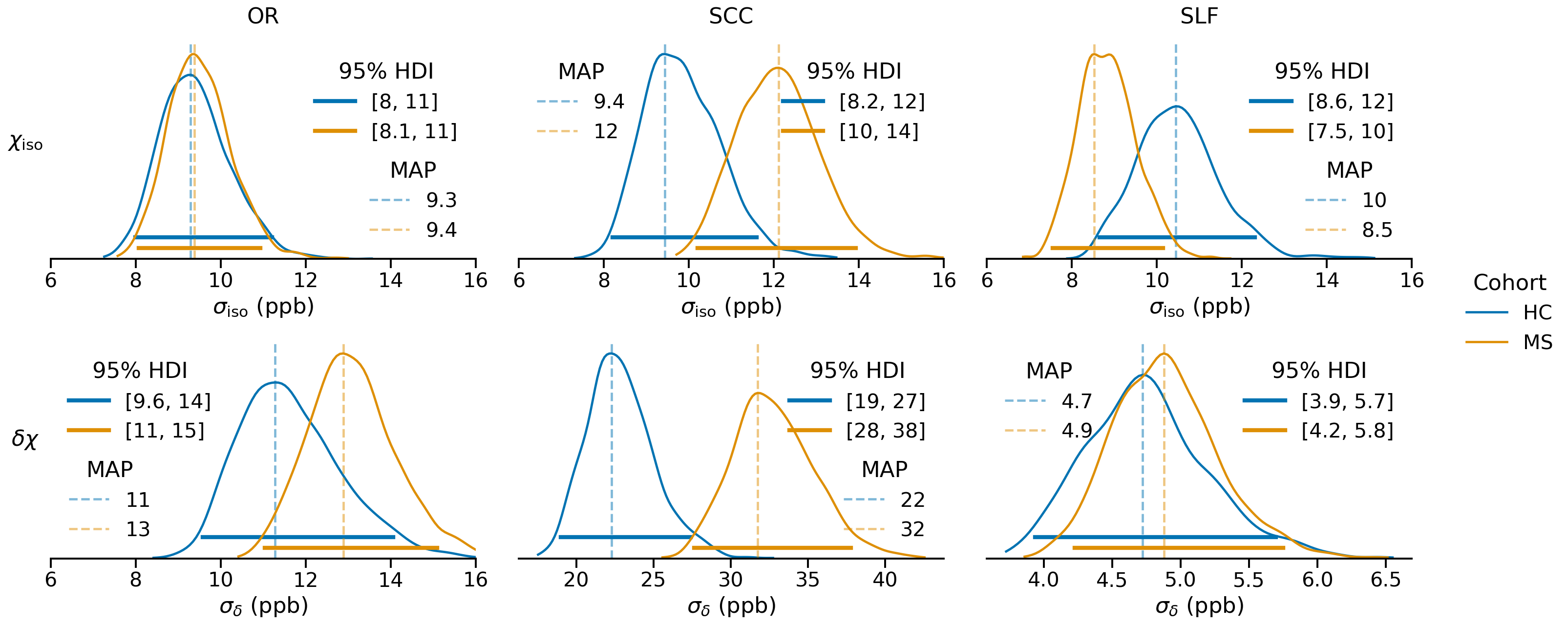


**Figure S3**: Marginal posterior distributions of cohort-level standard deviations $\sigma_{\text{iso}}^{\text{coh}}$ (top row) and $\sigma_{\delta\chi}^{\text{coh}}$ (bottom row). Dashed vertical lines represent the MAP estimate, while solid horizontal lines indicate the 95% HDI (i.e., credible interval of the estimated standard deviations).


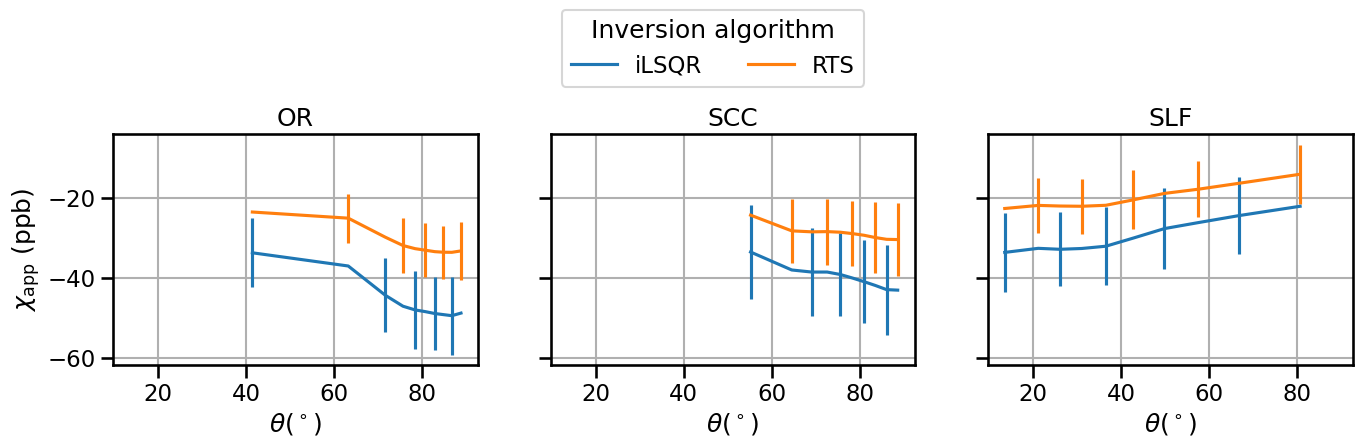


**Figure S4:** Orientation dependence of apparent magnetic susceptibility reconstructed with two different QSM inversion algorithms (iLSQR — iterative sparse linear equation and least-squares; RTS — rapid two step algorithm). Each line indicates the mean and two standard deviations across the cohort of healthy subjects (same aggregation as in Figure 2 of the main text; for clarity error bars are plotted for every other bin only).

The average trends in the healthy control cohort are consistent for the two inversion algorithms considered.


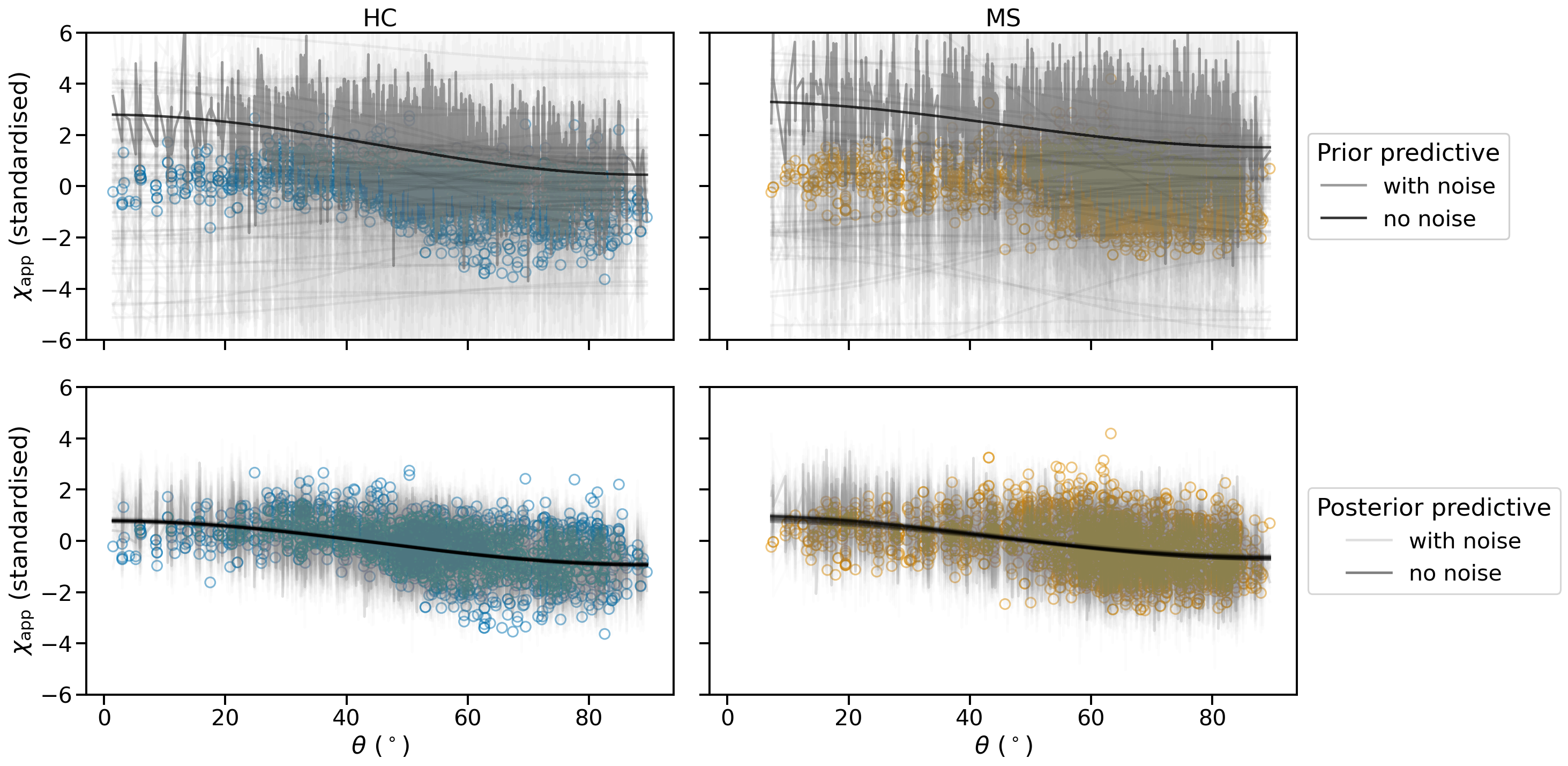


**Figure S5:** Predictive distributions of $\chi_{\text{app}}$ vs $\theta$ for the optic radiation in two random subjects, one from each cohort. Prior predictive (top row) illustrates 50 possible $\chi_{\text{app}}(\theta)$ curves consistent with the priors. Posterior predictive (bottom row) shows 50 curves produced by the posterior. The black lines represent the linear predictor for $\chi_{\text{app}}$, while the grey lines show the expected noise distribution. In each subfigure, one of the 50 predictions is highlighted (indistinguishable in the posterior predictive). All axes share the same scale.

Predictive distributions can be seen as simulations with a chosen parameter distribution. To construct the prior predictive distribution, these parameters are drawn independently from the priors defined in equations A1–12. The posterior predictive distribution, in turn, is constructed using the samples returned by the Markov chain Monte Carlo algorithm that approximates the posterior distribution. Note that the standard deviation of the noise observed per-subject $\sigma^{\text{subj}}$ is sampled like any other parameter and is subsequently part of the joint posterior distribution (although its marginal distributions are never shown in this work).

**Table S1**: Summary of the posteriors of the cohort-level means $\mu_{\text{iso}}^{\text{coh}}$ and $\mu_{\delta\chi}^{\text{coh}}$, given as the maximum a posteriori (MAP) estimate and the 95% highest density interval (HDI).

|  |  | Population mean | | | | | | |
| --- | --- | --- | --- | --- | --- | --- | --- | --- |
|  |  | $\mu_{\mathrm{iso}}$ (ppb) | | | | $\mu_{\delta\chi}$ (ppb) | | |
| ROI | Cohort | | MAP | 95% HDI | | MAP | 95% HDI | |
| OR | HC | | -54 | -56 | -51 | 27 | 24 | 30 |
| OR | MS | | -50 | -54 | -47 | 19 | 14 | 25 |
| SCC | HC | | -47 | -50 | -45 | 21 | 14 | 26 |
| SCC | MS | | -44 | -49 | -39 | 13 | 1.1 | 26 |
| SLF | HC | | -20 | -22 | -16 | -16 | -17 | -15 |
| SLF | MS | | -19 | -23 | -16 | -15 | -17 | -13 |

**Table S2**: Most prominent effects observed in the posterior of Equation 3. The 95% highest density intervals (HDI) in bold exclude 0 and as such indicate significance in this study.

| Parameter | ROI | MAP | 95% HDI |
| --- | --- | --- | --- |
| $\mu_{\delta\chi}^{\mathrm{MS}}-\mu_{\delta\chi}^{\mathrm{HC}}$ | OR | -0.6 | **[-1, -0.1]** |
| $\beta_{\mathrm{iso}}$ (ppb / 10 years) | OR | 3 | **[1, 5]** |
|  | SCC | 5 | **[3, 7]** |
| $\beta_{\delta\chi}$ (ppb / 10 years) | OR | -2 | **[-5, -0.3]** |
|  | SCC | -5 | **[-10, -1.1]** |
|  | SLF | 1.4 | **[0.5, 2]** |
| $\tau_{\delta\chi}^{\mathrm{MS}}$ (ppb / 10 years) | SCC | -12 | **[-22, -3.1]** |
| $\lambda_{\mathrm{iso}}^{\mathrm{MS}}$ (ppb / 10% lesion load) | OR | 2 | [-0.3, 4] |
|  | SLF | -2 | [-4, 0.6] |
| $\lambda_{\delta\chi}^{\mathrm{MS}}$ (ppb / 10% lesion load) | OR | -3 | **[-6, -0.5]** |
|  | SLF | 0.8 | [-0.6, 2] |
